# The heterogeneity of depressions: A phenomenological viewpoint

**DOI:** 10.1192/j.eurpsy.2023.21

**Published:** 2023-04-11

**Authors:** Giovanni Stanghellini

**Affiliations:** 1 Department of Health Sciences, University of Florence, Florence, Italy; 2 Centro de Estudios de Fenomenologia y Psiquiatria, Universidad ‘Diego Portales’, Santiago, Chile

**Keywords:** Depression, limit-situation, pre-morbid personality, subtypes, values

## Abstract

What is commonly referred to as “depression” indicates a heterogeneous complex disorder that includes distinct psychopathological forms. A more accurate classification can be achieved by assessing the individual experiences of depression and tracing back each of these forms to the specific vulnerable structure from which they emerge. Each of these vulnerable forms of existence can be characterized by focusing on their values. I identify four main prototypes. Homo melancholicus is impressive for their over-normality, extreme social adjustment, and conformism; their depressive decomposition is characterized by the experience of loss. The values of homo œconomicus are utility (every action must be directed toward production) and optimization (what costs more than it produces is a dead branch to be cut); their depressive decomposition is marked by insolvency. Homo dissipans’ values are excess (feeling animated by an inexhaustible drive to “pour out” of oneself, especially outside the limits of composure and reasonableness) and expenditure (an ethical attitude that gives its approval to excess and to its metamorphic and destructive power); their depressive decomposition is epitomized by inner incoherence and emptiness. The style of homo nevroticus form of existence is subjugated by the condemnation to limitation and the diktat of prohibition; their depressive decomposition is characterized by frustration and demoralization.

The term “depression,” used in the singular, is clinically misleading as it suggests a unitary and well-defined nosographic category, a symptom (depressed mood), or a phase of a disorder (major depressive episode). Instead, what is commonly referred to as “depression” indicates a heterogeneous complex phenomenon that includes distinct psychopathological forms. More accurate classifications, as for instance phenomenological ones, recognize discrete subtypes that can be identified on the basis of individual experiences of depression. Indeed, several (although not infinite) forms of depressive experiences exist. Some are characterized by guilt, others by anger, dysphoria, spleen, shame, emptiness, frustration, demoralization, exhaustion, and so forth. The mainstream approach to the diagnosis of depression mainly relies on “ticking boxes,” whose aim is neither an in-depth understanding of the patient’s personal experiences nor revealing previously unknown features of the patient’s condition. Rather, the aim of this approach is to assess those phenomena that are a priori deemed important as diagnostic indexes leading to the classification of the patient’s complaints and dysfunctions according to predefined – and, in the case of major depression, overinclusive – diagnostic categories. The main shortcomings of ticking boxes are “Procrustean errors” (stretching and trimming the patient’s symptomatology to fit criteria) [1] and “tunnel vision” (avoiding the assessment of phenomena not included in standardized interviews) [2].

In this paper, I outline a method for a more precise and detailed characterization of depression based on what I will call “drafting arrows” and “linking dots” [3]. In a nutshell, “drafting arrows” is constructing motivational diagrams connecting a given premorbid personality make-up with a given form of acute depressive decomposition via the corresponding pathogenic (traumatic) pre-depressive limit-situation. If we want to understand the forms taken by depression, we must trace back each of these forms to its specific anthropological matrix, that is, to the vulnerable structure from which they emerge. These structures – I assume – are axiotypes, that is, forms of existence characterized by a given moral value (what is important or “matters to” a given individual pre-structuring a worldview that establishes what is relevant and meaningful).

“Linking dots” means looking for structural links between the various aspects of a person’s experience, assuming that the manifold of phenomena of a given psychopathological condition have a meaningful coherence [4]. These structural links are to be looked for at each level of the motivational diagram – vulnerable anthropological matrix and acute depressive decomposition – and in the narrative links that connect each of the moments in the development of depression.

The contributions that phenomenological approach can make to the standard clinical one can be summed up as follows: taking the patient’s perspective rather than assessing the patient’s symptoms from a third-person perspective, recognizing and understanding the patient’s form of existence rather than simply diagnosing it, and remaining open to anomalies and individuality rather than being merely preoccupied with classification.

With all that in place, we can identify four main prototypes of depressive trajectories. I will name each of them according to the vulnerable anthropologic matrix they arise from: homo melancholicus, homo œconomicus, homo dissipans, and homo nevroticus (see Table 1).

**Table 1.** Anthropological forms, limit situations and clinical forms.

I must!	I want!	I can!	I cannot…
Homo melancholicus	Homo dissipans	Homo oeconomicus	Homo nevroticus
Conscientiousness orderliness hyper/heteronomia	Expenditure excess intensity	Performance utility optimization	Impossibility prohibition
Loss	Abandonment	Insolvency	Conflict
Guilt, ruin, illness	Feeling of emptiness, importent rage, persecutory rage	Exhaustion, bankruptcy, impossibility of power	Frustration, demoralization

The behavioral style of homo melancholicus [5] is impressive for their over-normality, extreme social adjustment, and conformism. It is the “I must!” personality, whose cornerstones are conscientiousness (the commitment to prevent guilt attributions and guilt feelings in the effort to feel accepted by others), orderliness (the fixation on harmony in interpersonal relationships and the need to avoid conflicts), and heteronomia (exaggerated receptiveness to external norms and impersonal motivation, referring to socially established criteria). The homo melancholicus pre-depressive limit-situation is characterized by a constant increase of the fixed tasks that overburdens their capacity to preserve a predetermined order. In such conditions, they are not capable of establishing a hierarchy of priorities and discriminate what can be momentarily left aside or postponed. Loss is the cipher of this gateway to depression and indeed they lag behind their commitments and feel guilty or lacking because of that. Consistent with this, acute depressive symptomatology also consists of themes of loss, that is, moral guilt, financial ruin, and physical infirmity, accompanied by reduced vital élan and emotional resonance.

The ethical imperatives of homo œconomicus – the “I can!” personality – are utility (every action must be directed toward a purpose, primarily to production) and optimization (what costs more than it produces is a dead branch to be cut). Being is equivalent to producing by adapting to the existing and by optimizing it. Every aspect of the existing is managed as a performance according to the logic of cost-effectiveness. Homo œconomicus are nevertheless vulnerable because, in their economy, self-construction becomes self-constriction. Their limit-situation is characterized by the failure of their own project. If they fail, they are solely responsible for their own failure – to them, an irremediable failure of the being-able-to-do (“I can”), therefore of the being-able-to-be (“I am”), since being is totally identified with doing. The psychopathological symptom ensuing from this failure is a state of psychic insolvency, that is, exhaustion, failure, loss of the project, the impossibility of the “I can!.” This distinguishes their decompensation from the characteristic symptoms of other forms of depression.

Homo dissipans is the opposite of homo œconomicus [6]. The core phenomena of this vulnerable form of existence – whose motto is “I want!” – are excess (feeling animated by an inexhaustible drive to “pour out” of oneself, especially outside the limits of composure and reasonableness) and expenditure (an ethical attitude that gives its approval to excess and to its metamorphic and destructive power). The homo dissipans’ credo is profitlessly dissipate their surplus of energy, escape into a world of pure intensities in which all forms – including identity – dissolve, and surrender themselves to transformation. Being is supposed to grow more when one accepts the risk of losing [7]. The social relationships of homo dissipans are a type of bonding with the Other in a daily limit-situation, destructive in nature. Especially erotic relationships are dominated by the absolute value of intensity that aspires to the fusion with the Other. Thus, they are intrinsically traumatic because this quest for excess always leads to the threshold of a feeling of dissatisfaction and emptiness that homo dissipans experiences as an (real or imagined) abandonment. Coherently, the main symptoms of their acute depressive decomposition are a painful experience of incoherence and inner emptiness, a feeling of uncertainty and inauthenticity in interpersonal relationships, often accompanied by feeling the victim of a persecutory Other and an excruciating sense of futility and inanity of life [8].

The style of homo nevroticus form of existence is subjugated by the moral imperative “I would like to but I can’t,” thus by the condemnation to limitation and the diktat of prohibition. They embody the quintessence of the human condition as finite freedom. Human beings, as finite freedom, are free within the contingencies of their finitude [9]. But within these limits, they are asked to make of themselves what they are supposed to become. This is what frightens homo neuroticus: their freedom, however limited, to realize themselves – to fulfill their destiny. Neurosis is the flight from one’s own freedom. Their limit-situation is conflict: the need to choose when faced with an alternative that brings anxiety. Homo nevroticus lacks the courage to choose. The outcomes of their incapacity to choose are discouragement, frustration, and demoralization. This is typically a chronic form of depression, with more subdued symptoms than other forms, but no less pernicious because it threatens to undermine one’s entire existence.

In conclusion, the efforts made by contemporary phenomenological research [10], also involving experts by experience, seem to be heading in the right direction, namely the characterization of the various forms of depression on the basis of first-person accounts, enriched not only by specialist but also by philosophical and literary sources. Also, the recognition of the patients’ values is increasingly significant for contemporary person-centered mental health care, with its emphasis on quality of life and development of self-management skills. The advantages of both clinical practice and empirical research are obvious. The treatment of patients suffering from “depression” cannot fail to take into account their personality structure and values, just as an appropriate pharmacological prescription must recognize the differences between the various clinical forms of depression. Finally, translational research requires valid psychopathological constructs, that is, rigorously defined on a phenomenal level, in order to identify the corresponding biological correlates.
